# Development of the Health Awareness and Behaviour Tool (HABiT): reliability and suitability for a Canadian older adult population

**DOI:** 10.1186/s41043-019-0206-0

**Published:** 2019-12-04

**Authors:** Gina Agarwal, Melissa Pirrie, Ricardo Angeles, Francine Marzanek, Jenna Parascandalo

**Affiliations:** 1 Department of Family Medicine, McMaster University, 100 Main St. W, DBHSC, Hamilton, Ontario Canada; 2 Department of Health Research, Methods, Evidence and Impact, McMaster University, Hamilton, Canada

**Keywords:** Questionnaire development, Older adults, Low income, Health behavior, Quality of life, Healthcare utilization, Health knowledge, Health literacy

## Abstract

**Background:**

Determining the effectiveness of community-based health promotion and disease prevention programs requires an appropriate data collection tool. This study aimed to develop a comprehensive health questionnaire for older adults, called the HABiT, and evaluate its reliability, content validity, and face validity in assessing individual health-related items (e.g., health status, healthcare utilization) and five specific scales: knowledge, current health behaviors (risk factors), health-related quality of life (HRQoL), perceived risk and understanding, and self-efficacy.

**Methods:**

Iterative survey development and evaluation of its psychometric properties in a convenience sample of 28 older adults (≥ 55 years old), half from a low-income population. Following item generation, the questionnaire was assessed for content validity (expert panel), face validity (participant feedback), internal consistency of each scale (Cronbach’s alpha), and test-retest reliability for each item and scale (Pearson’s *r* and phi correlations, as appropriate).

**Results:**

Questions were drawn from 15 sources, but primarily three surveys: Canadian Community Health Survey, Canadian Diabetes Risk Questionnaire (CANRISK), and a survey by the Canadian Hypertension Education Program. Expert consensus was attained for item inclusion and representation of the desired constructs. Participants completing the questionnaire deemed the questions to be clear and appropriate. Test-retest reliability for many individual items was moderate-to-high, with some exceptions for items that can reasonably change in a short period (e.g., perceived day-to-day stress). Of the five potential scales evaluated, two had acceptable internal consistency (Cronbach’s alpha ≥ 0.60) and a subset of one scale also had acceptable internal consistency. Test-retest reliability was high (correlation ≥ 0.80) for all scales and sub-scales.

**Conclusions:**

The HABiT is a reliable and suitable comprehensive tool with content and face validity that can be used to evaluate health promotion and chronic disease prevention programs in older adults, including low-income older adults. Some noted limitations are discussed. Data collected using this tool also provides a diabetes risk score, health literacy score, and quality-adjusted life years (QALYs) for economic analysis.

## Introduction

A key element when evaluating individual-level health promotion and disease prevention programs in the community is accurately measuring health knowledge (e.g., risk factors for diabetes), current health behaviors, perceived risk and understanding (e.g., concern about chronic disease risk, understanding the importance of changing the health behavior), self-efficacy to improve health behaviors, current health status, and healthcare utilization [1–3]. However, finding the right tool that is context-specific, valid, and reliable is challenging.

In planning the evaluation of the Community Paramedicine at Clinic (CP@clinic) program, it was identified that each of the health-related items listed above needed to be measured in a population of Canadian older adults. CP@clinic is a community-based health program for older adults (aged 55 and over) living in low-income housing apartment buildings [4, 5]. The weekly, one-on-one drop-in program is open to all building residents and there is no cost to attend. Administered by the local paramedic service, whether municipal or regional, CP@clinic assesses participants’ risk factors for cardiovascular disease (CVD), diabetes, and falls, and delivers health education/promotion specific to participants’ risk factors. Participants are then referred to community resources to help them modify their risk factors and reports are sent to their family physicians. The underlying theory for CP@clinic is that the program will improve health knowledge, awareness, and perceived risk of CVD and diabetes, and also improve self-efficacy to change these risk factors leading to a change in health behavior [5, 6]. Health behavior changes will in turn lead to improved health, better quality of life, and decreased use of emergency health services and hospitalizations due to CVD, diabetes, and falls [5, 6]. This was demonstrated in a pilot project of the CP@clinic program [6]. In order to evaluate this theory, a tool was needed that non-health professionals (e.g., trained research staff) could use to accurately assess this health promotion and chronic disease program in a low-income older adult population and within a reasonable time frame (approximately 20 min).

Health-related questionnaires are typically either comprehensive (i.e., general health surveys) [7–12] or focused on one specific health issue or behavior [13–19]. The Canadian Community Health Survey (CCHS) [7] is a well-known comprehensive, evidence-based survey that incorporates many of the domains covered by CP@clinic and is used widely with a Canadian population. However, it does not include questions on self-efficacy or perceived risk and goes beyond the scope of CP@clinic by including topics such as gambling, sexual health, and sun behavior; as a consequence, it is quite lengthy (50 min average completion time [8]) and unfeasible for administration in this context. Similarly, the CCHS - Healthy Aging questionnaire is very comprehensive (37 modules) [9], but was found to be exceedingly long in pilot testing (needed to be split into two parts) and took an average of 62 min to complete in the final data collection [10]. The CCHS - Healthy Aging also did not include all of the topics required for evaluating the impact of a health promotion and disease prevention program, such as knowledge of risk factors and self-efficacy to change. CCHS has not repeated the Healthy Aging questionnaire since 2009. The Canadian Health Measures Survey is a general health survey that includes physical measures [11]; this survey requires blood draws and specialized equipment (e.g., spirometer), which would not be relevant or practical in the CP@clinic context. Other comprehensive surveys developed for non-Canadian populations, such as the National Health Interview Survey [12], have the same limitations as the CCHS [7]. Therefore, there were no existing comprehensive surveys identified that covered all of the desired items and could feasibly be used to evaluate the CP@clinic program within a 20-min interview.

Questionnaires that measure specific topics like sedentary behavior [13], quality of life [14, 15], diabetes attitude and behavior [16], diet [17], or lifestyle risk factors [18] have been developed, but they have not been tested for reliability and validity in a Canadian older adult population. Also, since these questionnaires are topic-specific, they tend to be more in-depth, with many questions on a single topic. Combining multiple complete, topic-specific questionnaires would result in a tool that is too long and impractical to administer. One notable exception is the Canadian Diabetes Risk (CANRISK) questionnaire [19], a validated questionnaire that assesses risk factors specific to diabetes in a Canadian population. Since the CANRISK questionnaire was designed to calculate a diabetes risk score using a minimal set of questions, it covers multiple topics (e.g., diet, physical activity) using a single, representative question for each risk factor [19] and can be feasibly integrated, in full, within a larger questionnaire.

Given that there are many existing questionnaires but none that satisfied the requirements of the CP@clinic program, the research team decided to adapt and combine selected tools into a comprehensive, multidimensional questionnaire, called the Health Awareness and Behaviour Tool (HABiT), tailored for older adults in Canada and appropriate for a low-income population. This paper describes the questionnaire development and the evaluation of its validity and reliability in assessing each health-related item (e.g., health status, healthcare utilization) and five specific scales: knowledge, current health behaviors (risk factors), health-related quality of life (HRQoL), perceived risk and understanding, and self-efficacy.

## Methods

### Questionnaire development

The standard method for iterative questionnaire development and validation was followed; this started with item generation and content validation, followed by evaluating face validity, internal consistency, and test-retest reliability [20]. The survey was intended to be an interviewer-led survey for ease of administration and completeness.

Item generation: The initial questionnaire items were gathered from multiple surveys that are frequently used in Canadian settings or were topics relevant to CP@clinic. Specifically, these topics were knowledge (CVD and diabetes); current health behaviors (physical activity and sedentary behavior, diet, smoking, alcohol use, stress); health status (e.g., hypertension, diabetes risk); perceived risk and understanding (e.g., concern about chronic disease risk, understanding the importance of changing the health behavior); self-efficacy; HRQoL; healthcare utilization; and, health literacy. Potential domains were identified by the expert panel according to literature on health promotion and disease prevention theory. The panel prioritized questions that were used in the Canadian population as that was our population of interest. They also used questions from surveys that were commonly used in health research and community health services, such as the CCHS. The panel also used questions from tools that used the constructs in which they were interested. The final selection was based on consensus of the expert panel. These items were compiled into a questionnaire, which we called the Health Awareness and Behaviour Tool or HABiT.

Content validity: The compiled questionnaire was presented to five content experts (a family doctor, public health nurse, public health doctor, researcher, and paramedic) to assess if the desired domains were appropriately covered by items included in the questionnaire. Items were added and modified based on the opinions of the content experts. The expert group met together and any differences were resolved by consensus.

Face validity, internal consistency, and test-retest reliability: These three measures were evaluated in a series of assessments with two groups of participants. The first group of participants was asked to complete the questionnaire and provide feedback on whether the questions measured the items of interest, whether the questions were clear and easy to answer, and whether there were specific modifications needed (face validity). Participant responses were tested for internal consistency using Cronbach’s alpha. When appropriate, item reduction was conducted to improve internal consistency within the questionnaire. Finally, a second group of participants was asked to complete the questionnaire twice, 2 weeks apart, to evaluate test-retest reliability. This second group also provided additional feedback on face validity.

### Participants

There were two groups of respondents who participated in the questionnaire evaluation. Informed consent was obtained from all participants. All participants were 55 years and older, which is the main inclusion criteria for the CP@clinic program. Convenience sampling was utilized to recruit participants specifically for this study (i.e., they were not a sample of CP@clinic participants). The initial questionnaire was tested in the first group of participants, refined, and then tested in the second group. Our sample size was calculated to be between 15 and 20, based on a conservative estimate for the number of items per domain (*k* = 10), and power of 80% at an alpha of 0.05, aiming for a Cronbach’s alpha of 0.65 [21]. The first group of participants was recruited through study investigators as individuals who were known, over 55 years of age, and willing to complete the survey. The second group of participants was invited to participate from the buildings in which the CP@clinic program took place, though the survey completion was not actually part of the CP@clinic program.

### Statistical analysis

Analysis was conducted for each questionnaire item individually and for five potential scales (knowledge, current health behaviors, perceived risk and understanding, HRQoL, and self-efficacy). The questions under each scale were assessed for convergence using Cronbach’s alpha. An acceptable alpha of > 0.60 was targeted for each scale since the questions measure different aspects of each scale and some scales can be considered multidimensional [20, 22, 23]. Items were considered for exclusion from a scale based on item to total correlation (< 0.2) and the degree of improvement in the alpha if item was removed. The final decision to remove any items was based on consensus between the researchers, specifically considering whether the alpha level was acceptable and information was not lost by removing the item from the questionnaire. For test-retest reliability of the binary items, phi coefficient was used to evaluate the correlation between the item’s scores measured twice, 2 weeks apart. Similarly, for test-retest reliability of continuous items, Pearson’s correlation coefficient (*r*) was used to determine the correlation between each score, as measured 2 weeks apart.

## Results

### Participants

Two groups of participants (*n* = 28 with 13 in group 1 and 15 in group 2) were recruited to complete the questionnaires and assess face validity. The same completed questionnaires were used for evaluating internal consistency and test-retest reliability. The two groups of participants were significantly different from each other with respect to age and education level (see Table 1). Over 50% of the participants in group 1 were over 60 years old compared with 40% in group 2. Also, 38% percent of group 1 participants had high school education or less, compared with only 20% of group 2 participants. The implications of these differences will be discussed throughout the remainder of this paper. There were no missing data on the questionnaire for both groups, with the exception of cases where the respondent opted to provide their pants size instead of a waist measurement.

**Table 1:** Participant profile

Socio-demographic variables	*n* (%)	
	Group 1 (*n* = 13)	Group 2 (*n* = 15)
Age		
≤ 59	6 (46.2)	9 (60.0)
60–69	2 (15.4)	5 (33.3)
> 70	5 (38.4)	1 (6.7)
Gender (female)	10 (76.9)	10 (66.7)
Marital status		
Married	9 (69.2)	11 (73.3)
Single	1 (7.7)	0
Widowed/divorced/separated	3 (23.1)	4 (26.7)
Education		
High school or less	5 (38.5)	3 (20.0)
Some college or university	0	6 (40.0)
University or college degree	8 (61.5)	6 (40.0)

### Item generation

Items in the HABiT questionnaire were derived from multiple sources; please see Table 2 for an overview of the questionnaire content and the Additional file 1 for the full questionnaire and sources.

**Table 2:** Test-retest reliability of individual items within the HABiT

Section	Items	Correlation coefficient (*r* or φ)
Demographics	Name, date of birth, postal code, ethnicity, marital status, education, employment status, income category	-
Self-reported health status (SRHS) and health-related quality of life (HRQoL)	SRHS	0.74
	HRQoL (mobility, self-care, usual activities, pain/discomfort, anxiety/depression, health status)	0.56–1.00
Knowledge	19 statements on CVD and diabetes	1.00
Current health status	Diagnosed conditions (heart problems, diabetes, stroke, hypertension, high cholesterol)	0.70–1.00
	Monitoring (blood pressure, cholesterol, blood sugar)	0.29–0.65
	Physical measures (height, weight, waist circumference)	0.90–0.95
	Diabetes-related medical history (birth to large baby, family history)	0.42–1.00
Current health behaviors	Physical activity (30 min daily per week; weekly mild, moderate, and strenuous physical activity)	0.63–1.00
	Sedentary behaviors (time spent on a computer, watching television, reading)	0.64–0.74
	Diet (fruit and vegetable consumption, monitoring weight, salt intake, and frequency of complex carbohydrate, fatty food, and sugar food intake)	0.42–0.78
	Tobacco use (smoking status, quantity smoked per day, years since quit, intention to quit)	0.94–1.00
	Alcohol use	
	• Weekly consumption	0.89
	• Binge drinking	0.22
	Stress (ability to handle an unexpected crisis, day-to-day demands, sources of stress)	0.02–0.32
Healthcare utilization and access	Availability of healthcare in community	0.76
	Sources of advice (sick, heart health, diabetes)	0.58–1.00
	Healthcare utilization (family doctor, EMS, walk-in clinic)	0.42–1.00
Health behavior change	Perceived risk and understanding (concern, understanding, importance)	0.59–0.97
	Intent to change	0.78
	Self-efficacy to change (physical activity, fruits and vegetables, alcohol use, smoking, stress)	0.40–1.00
Health literacy	Newest vital signs score	-

The survey foundation was established using items from three questionnaires: CCHS [7], CANRIS K[19], and a hypertension management survey developed and used by researchers from the Canadian Hypertension Education Program (CHEP) [24], which was partially based on the work of Petrella and colleagues [25]. The expert panel reviewed these three questionnaires to determine the elements measuring current health status, health system access and utilization, and current health behaviors that were most relevant for older adults. Redundant questions were eliminated during this phase. All questions from the CANRISK questionnaire [19] were retained to ensure that a diabetes risk score could be calculated from HABiT responses. Gaps not covered by these core questionnaires (e.g., health literacy, self-efficacy), were filled by adding and adapting select questions from other questionnaires. Items included in each section of the questionnaire have been described below.

Demographics: The HABiT collects full name, postal code, date of birth, maternal and paternal ethnicity, marital status, employment status, and annual income. The questions and response options were carefully selected to fulfill three purposes: (1) facilitating linkage with administrative healthcare utilization databases, (2) matching the CANRISK questionnaire [19] so a diabetes risk score can be calculated, and (3) matching common population-level surveys (e.g., CCHS, Canadian census) to allow comparisons between populations. The cross-population comparisons would allow health services researchers to extract data in multiple populations that can be of use in health service planning, and it is not possible to replicate large national survey methods for such smaller comparison-based samples.

Self-reported health status (SRHS) and HRQoL: This section begins with a single question on SRHS from the CCHS [7], “In general, would you say your health is...” (five response options from poor to excellent). This is followed by the EQ-5D-3 L [26], a validated questionnaire developed by the EuroQol Group that measures HRQoL and can be used to calculate quality-adjusted life years (QALYs) for economic evaluation [27]. The EQ-5D-3 L, which has been applied previously in a paramedic setting in Ontario [28], has two components: (1) a Likert-scale of 5 items (mobility, self-care, usual activities, pain/discomfort, and anxiety/depression), each with three response options (no problems to extreme problems); and, (2) and a visual analog scale of health status on the current day, presented as a thermometer from 0 (worst imaginable health state) to 100 (best imaginable health state). These questions were unaltered from the validated EQ-5D-3 L questionnaire [27].

Knowledge: This section contains 19 short statements about CVD and diabetes; for each statement, respondents indicate their response on a 5-point scale (definitely true to definitely false). Questions regarding CVD knowledge were drawn primarily from the CHEP survey [24] and a chronic disease knowledge questionnaire [29]. Diabetes knowledge questions were drawn from the CHEP survey [24], the Diabetes Knowledge Questionnaire [30], and a chronic disease knowledge questionnaire [29], with the exception of three questions. The three exceptions were CVD knowledge questions that were replicated for diabetes (e.g., “High blood pressure can cause other serious health problems” was replicated as, “Diabetes can cause other serious health problems”).

Current health status: For CP@clinic evaluation, it was important to determine the baseline health condition of older adults living in social housing, both in buildings where the CP@clinic program would be implemented, as well as in the control buildings. Accordingly, the HABiT contains 11 questions assessing the respondent’s current health including diagnosed health conditions (e.g., diabetes, stroke, hypertension), health monitoring (blood pressure, cholesterol, and blood sugar), physical measures (height, weight, and waist circumference), and diabetes-related medical history (given birth to a large baby, blood relatives with diabetes). Questions in this section were drawn from the CCHS [7], CHEP [24], and CANRISK [19] surveys. Self-reported height and weight are collected to calculate body mass index, and waist measurement is taken using a measuring tape by the individual or a survey administrator (instructions for taking the waist measurement are included within the survey text). Both of these items are used in the CANRISK score calculation [19]. The HABiT includes an option for pants size to be provided as an estimate of waist circumference; this is not an option in the CANRISK. This option was included to facilitate imputation for research purposes and to minimize missing data due to our previous experiences administering the CANRISK in a similar population. In an interviewer-administered scenario, the option to provide pants size is only offered after the respondent declines having their waist circumference measured.

Current health behaviors: The HABiT collects data on six health behaviors related to chronic disease prevention and HRQoL: physical activity, sedentary behavior, diet, tobacco use, alcohol use, and stress.

*Physical activity*. Physical activity is first assessed using a single question from the CANRISK that functions as an indicator for overall physical activity to calculate diabetes risk [19]. Specifically, it asks if the individual engages in at least 30 min of physical activity each day of the week, such as brisk walking. Next, the Godin Leisure-Time Exercise Questionnaire [31] (GLTEQ) was incorporated as a more sensitive measurement tool that can classify individuals into activity levels and demonstrate health behavior changes. The GLTEQ includes three questions on how many times in a typical week the respondent exercises for more than 15 min at mild, moderate, and strenuous intensities [31]. The GLTEQ includes example activities for each intensity, but they include many activities and sports that are not common among older adults. Following the lead of previous researchers [32, 33], the example activities were replaced by other activities that are more common among older adults and have the same metabolic equivalent value based on the latest compendium of physical activities [34], for example, gardening (mild), water aerobics (moderate), and fast stair climbing (strenuous). In adapting physical activity measures for seniors, researchers found that changing the examples was insufficient and seniors were uncomfortable with the standard order of these questions (strenuous, moderate, and then mild) since they rarely participate in strenuous activities and seldom in moderate activities [32]. Instead, a validated physical activity questionnaire for seniors found that asking about mild activities first was more appropriate and representative of the behaviors in this population [32]. Therefore, the physical activity questions in HABiT progress from mild to strenuous intensity.

*Sedentary behaviors*. Three questions measuring sedentary behaviors were included from the CCHS, asking the respondent how many hours in a typical day of the week they spend on a computer (e.g., playing games), watching television/videos, and reading [7].

*Diet*. The CANRISK uses a single question on fruit and vegetable consumption (response options: every day, not every day) in the risk score calculation [19]. A very similar question is included in the CHEP survey [24], but with a greater range of response options for those who do not consume fruit and vegetables daily (i.e., number of times per week they are consumed). Therefore, the CANRISK question was maintained in the HABiT for validity in scoring the CANRISK, but the expanded list of response options from CHEP was added to better understand the profile of fruit and vegetable intake among older adults living in social housing. Additional questions on diet were drawn from the CHEP survey, including the number of portions of fruit/vegetables consumed each day (response options expanded to match the 2011 Canada’s Food Guide) [35], whether the respondent monitors their food intake to maintain a healthy weight, frequency of complex carbohydrate intake (e.g., breads), frequency of fatty food intake, frequency of sugary food intake, and the addition of salt to food while cooking or at the table [24].

*Tobacco use*. This section begins with a single question on smoking status adapted from both the CCHS [7] and CHEP [24] surveys to determine if the respondent is a daily smoker, occasional smoker, former smoker, or non-smoker. For current smokers, they are asked the quantity of cigarettes, cigars, and pipes smoked per week (based on CHEP). Current smokers are also asked about quitting intentions: no plans to quit, have thought about quitting, have a plan to quit smoking, or have initiated a plan to quit smoking. These response options are based on the Stages of Change theory [36] and Health Canada’s Five Stages to Quitting [37]. Respondents who have quit smoking are asked the number of years since they quit and the quantity of cigarettes, cigars, and pipes smoked per day before they quit; both questions are from the CHEP survey [24].

*Alcohol use*. Following Canada’s Low-Risk Alcohol Drinking Guidelines [38], two questions on alcohol use were included: one on weekly consumption (from CHEP) [24] and one on binge drinking (from CCHS) [7]. The section begins by defining “one drink” according to the guidelines [38]. Next, respondents are asked the number of drinks consumed in an average week with five response options ranging from “non-drinking/rarely/have stopped drinking” to “More than 15,” including categories that align with the recommended weekly consumption maximums for both men and women. Subsequently, respondents are asked how often in the past 12 months have they had five or more drinks on one occasion, with five response options ranging from “Never or Less than once per month” to “More than once per week.”

*Stress*. Based on the CCHS survey [7], three questions were included about stress: the respondent’s ability to handle unexpected problems or a crisis (5-point scale), their ability to handle day-to-day demands (5-point scale), and finally, contributors to their daily stress (15 response options, including “other” and the opportunity to specify their other sources of stress).

Healthcare utilization and access: This section includes 10 questions. Based on the CCHS survey [7], respondents are asked to rate the availability of healthcare in the community, where they usually go when sick or need advice (e.g., walk-in clinic, TeleHealth), whether they have a regular family doctor, and the reason why they did not seek care when sick, if applicable. Based on the CHEP survey [24], respondents are asked about where they get information on keeping their heart healthy and preventing diabetes. The response option of whether there were paramedics in their building was added to incorporate the CP@clinic program that would be evaluated. Finally, while the CHEP survey asks respondents how many times they have been admitted to hospital in the last 12 months, this question was adapted for the HABiT to assess number of visits to their regular family doctor, number of calls to EMS, and number of visits to a walk-in clinic in the previous 12 months.

Health behavior change: Three separate but interrelated constructs comprise this section: (1) perceived risk and understanding; (2) intent to change; and, (3) self-efficacy to change.

*Perceived risk and understanding*. Guided by the Health Belief Model scales [39] and the Brief Illness Perception Questionnaire [40], the HABiT includes seven 7-point Likert-type scales evaluating the respondent’s degree of concern regarding high blood pressure and diabetes (“not at all concerned” to “extremely concerned”), degree of understanding regarding their risk of high blood pressure and diabetes (“do not understand” to “complete understand”), and degree of importance for increasing fruit and vegetable intake, decreasing intake of foods high in salt, and increasing physical activity (“not at all important” to “extremely important”).

*Intent to change*. Three intent to change questions were drawn from the CCHS [7]. The respondent is asked if there is anything they intend to improve in the 12 months and, if yes, are presented with nine response options of health behaviors they could improve (e.g., lose weight, change diet), including “other” and the opportunity to specify. For respondents who are current smokers, they are also asked if they seriously intend to quit smoking within the next 6 months.

*Self-efficacy to change*. Guided by the Stanford Chronic Disease Self-Efficacy Scales [41], the HABiT includes five 7-point Likert-type scales (“not at all confident” to “extremely confident”) evaluating the respondent’s confidence to improve physical activity, increase intake of fruits and vegetables, reduce alcohol intake, quit smoking, and reduce stress.

Health literacy: The final section of the HABiT is the complete Newest Vital Sign [42]; a short, validated health literacy assessment tool. The respondent is presented with a nutrition label for ice cream and asked a series of six questions evaluating their ability to interpret the information presented (e.g., serving size, calories per serving, ingredient list).

### Content validity

After compiling the questionnaire items, the preliminary tool was presented to content experts who made suggestions regarding the questionnaire content, organization, structure, and appearance. After the final review for content validity, all experts were in agreement that target health-related topics and domains were satisfactorily measured by the items included in the questionnaire and no additional questions were added to the instrument.

### Face validity

The first group of participants was asked to complete the questionnaire and provide feedback on whether they felt each of the items and topics targeted by the questionnaire were addressed and if the questions were clear and unambiguous. The participants agreed that the questionnaire did assess the intended areas; however, a few participants found some questions and response options unclear. The questionnaire was subsequently revised based on the comments and suggestions. Next, the second group of participants was asked to complete the questionnaire and provide additional feedback. There were no additional changes suggested by the second group of participants for questions or response options; they felt the questions were clear and accurately addressed the intended topics.

### Internal consistency

For sections of the HABiT evaluated as potential domains (e.g., knowledge about CVD and diabetes), Cronbach’s alpha was initially calculated based on the questionnaires completed by the first group of participants (see Table 3). The initial alpha for “Knowledge about CVD and Diabetes” as a single scale was 0.54. Some questions had low item-to-total correlation (< 0.20); by removing two items and segregating the remaining items into two subdomains (“Risk Factors” and “Common Misconceptions”), the alpha improved. The “Risk Factors” subdomain achieved an acceptable alpha (> 0.60); however, “Common Misconceptions” had an alpha of < 0.60. The Cronbach’s alpha was finally calculated as 0.70 for group 2 for “Knowledge about CVD and Diabetes” and 0.55 for “Common Misconceptions.” The reason for the low alpha may be due to the low number of items, low number of participants, and/or the variability in responding to these questions. Since these items (i.e., common misconceptions) were important for evaluating an intervention that improves knowledge of participants regarding CVD and diabetes, it was decided to retain these items in the HABiT questionnaire but segregated as a subdomain with this limitation noted.

**Table 3:** Internal consistency and test-retest reliability of domain scales

Domains	Subdomains (no. of items)	Cronbach’s alpha		Correlation Coefficient (*r* or φ)
		Group 1	Group 2	
Knowledge about CVD and diabetes	Risk factors (15)	0.77	0.70	1.00
	Common misconception (3)	0.34	0.55	1.00
Current health behavior	No subdomains (13) Multiple behaviors (sedentary lifestyle, diet, alcohol, smoking) analyzed as single scale	0.40	0.74	0.85
Health-related quality of life (HRQoL)	No subdomains (5)	0.96	0.58	0.88
Perceived risk and understanding	No subdomains (7)	0.88	0.88	0.96
Self-efficacy	No subdomains (5)	0.68	0.78	0.86

In developing the “Current Health Behaviors” domain, all behaviors were analyzed together as a single scale since there were a limited number of questions for each behavior (i.e., 2–4 questions). Ability to handle stress (2 items) was removed from the scale since the item-total correlation was low. The Cronbach’s alpha was 0.4 in group 1 and 0.74 in group 2. Although the alpha was acceptable in group 2 (younger, higher education), the findings suggest that it would be better to consider each question under this domain as individual items and not as a scale when using this instrument.

The “HRQoL” domain includes the five HRQoL items (e.g., pain/discomfort) and the visual analog health status scale that comprise the EQ-5D-3 L [26]. Although validation studies commonly evaluate each item of EQ-5D-3 L separately [14, 15], the items were analyzed as a single scale to test if this could be used as another global measure. The results were not consistent between the two groups of participants. The alpha was very high in group 1(alpha = 0.96) but not acceptable in group 2 (alpha = 0.58). Further evaluation shows that the mean and variance in group 1 were 14.1 and 6.1, respectively, compared with 14.2 and 1.1 in group 2. Group 1 had more diverse responses, which coincides with the participant profile (more diverse education level and age). The results therefore may suggest that the domain should be used with caution when evaluating a heterogenous group. Alternatively, 5-digit health state vectors can be derived from the first five questions of the EQ-5D-3 L, each with three response options, for example, 11321. Next, QALYs associated with each vector can be assigned based on the available country-specific value sets [27, 43]. Numerous valuation studies have been conducted to translate the EQ-5D health state vectors into QALYs, a broad measure of HRQoL [27, 44].

Both “Perceived Risk and Understanding” and “Self-efficacy” yielded acceptable alphas in both groups 1 and 2 (0.68–0.88). The questions and responses were retained without changes.

### Test-retest reliability

The second group of participants was asked to complete the refined HABiT questionnaire twice, 2 weeks apart. This version of the HABiT did not include the health literacy measure and therefore no data is available on the test-retest reliability in this sample. The correlation coefficients showed moderate-to-high correlations for many of the items and domains between the two time periods (see Tables 2 and 3), indicating adequate test-retest reliability. Test-retest correlations were low for measures that can readily change within 2 weeks without intervention (e.g., feelings of stress) or due to prompting by the first questionnaire administration (e.g., blood pressure checks).

The final survey version took 20 min to complete and was interviewer-led.

## Discussion

The HABiT was developed to measure health knowledge, SRHS, HRQoL, current health status, current health behaviors, healthcare utilization and access, health behavior change (e.g., intent, self-efficacy), and health literacy in older adults. It was also intended to provide scales for five domains: (1) knowledge about CVD and diabetes, (2) current health behavior, (3) HRQoL, (4) perceived risk and understanding, and (5) self-efficacy. This work was completed in the context of developing a measure to evaluate health interventions with older adults, specifically CP@clinic. While we did not evaluate construct validity, we conclude that the HABiT tool is reliable, has internal consistency, and has both content and face validity in this population. Test-retest reliability was moderate-to-high, except for the individual items under health monitoring and stress, which can reasonably be expected to change within a 2-week time period. We also recognize that the HRQoL domain, comprising of EQ-5D-3 L, is already validated in this population. Our purpose with including this domain was to provide descriptive information of its administration in this population.

For scales within the HABiT, the “Perceived Risk and Understanding” domain, the “Self-Efficacy” domain, and the risk factor subdomain within “Knowledge about CVD and Diabetes” had good psychometric properties; therefore, these scales can be used to compare populations (e.g., control and intervention) as well as evaluate changes in a sample population (e.g., pre-post intervention). The “HRQoL” domain, combining the five dimensions of EQ-5D-3 L, did not yield a consistent result as a scale and each item should be evaluated individually or should be evaluated using the vector and QALY approach [44]. Finally, internal consistency for the “Current Health Behavior” domain was low in group 1. Considering that health behavior is multidimensional and each behavior had 2 to 4 questions, this study suggests that these behaviors be analyzed as individual items and not summarized as a scale. The test-retest reliability for individual health behavior items was high, indicating that the responses were stable, which supports item-level analysis measuring health behavior with the HABiT.

A strength of the HABiT tool developed in this study is its comprehensiveness within a relatively short questionnaire; it can be completed within 20 min compared with other comprehensive surveys that take 50–60 min [8, 10]. The aim was to measure numerous health-related dimensions with sufficient detail to adequately provide a snapshot of that dimension without including so much depth that the tool was too cumbersome to practically implement. Also, the survey tool was both tailored to and tested in older adults, not the general population. There were no other comprehensive health surveys (i.e., not focused on a specific behavior or disease) identified in the literature that were tailored to this population, covered the required topics, and were not lengthy. As the number of older adults quickly grows in Canada and abroad, these individuals are expected to be the focus of an increasing number of health-related interventions and having an appropriate tool available is critical. Additionally, in our development of the tool, half our development sample consisted of low-income older adults, in whom education and health literacy are lower [45]. This population would make an ideal target for health-related interventions. Thus the HABiT is not only appropriate for an older adult population, but can be used appropriately in a low-income older adult population. Finally, the complete CANRISK questionnaire was incorporated into the HABiT, allowing the respondent’s 10-year diabetes risk score to be calculated.

However, there are some limitations to the HABiT. Some items within the HABiT that can reasonably change day-to-day or within the 2-week test-retest time period (e.g., having their BP checked) demonstrated low test-retest reliability. Therefore, these items should be used with caution and not used to measure change over time. Also, the established health literacy measure at the end of the survey was not included in the test-retest evaluation. This measure is an optional, stand-alone component of the HABiT and details on its internal and test-retest reliability can be found in the literature [42]. Another limitation is the fact that some questions were drawn from the 2011 Canada’s Food Guide which has since been updated (2019) and no longer includes serving sizes [46]. It is important to note that the original questionnaires from which the questions were drawn will have had limitations; these will continue in the HABiT as well. Information collected for the HABiT is self-reported, even for interviewer-led questioning, therefore must be interpreted with caution, as with any self-reported data. The HABiT could reasonably be applied to older adults in Canada who speak English, though may not be fully applicable beyond this group without further testing. Lastly, the sample size was relatively small. It does however meet the minimum sample size requirement for subdomains that were measured. Further testing can be done with a larger sample of the intended population.

## Conclusions

The HABiT is a useful tool for measuring multiple health-related dimensions in older adults in the community, as well as low-income older adults. While each item can be considered individually, three of the scales developed (two domains and one subdomain) were found to be psychometrically sound for measuring single time points and changes over time. Additional research is needed to better understand the validity and reliability of this survey with different age categories (e.g., 85 years and older) and in different older adult populations in Canada (e.g., minority populations).

## Supplementary information

**Additional file 1.** HABiT Questionnaire.

## Data Availability

The data are available from the corresponding author upon reasonable request.

## Abbreviations

CANRISK: Canadian Diabetes Risk Questionnaire
CCHS: Canadian Community Health Survey
CHEP: Canadian Hypertension Education Program
CP@clinic: Community Paramedicine at Clinic
CVD: Cardiovascular Disease
EQ-5D-3 L: EuroQol 5 Dimensions—3 Levels
GLTEQ: Godin Leisure-Time Exercise Questionnaire
HABiT: Health Awareness and Behaviour Tool
HRQoL: Health-Related Quality of Life
QALY: Quality-Adjusted Life Year
SRHS: Self-Reported Health Status
