# Supplementary material for: Development of the Health Awareness and Behaviour Tool (HABiT): reliability and suitability for a Canadian older adult population
Source: J Health Popul Nutr. 2019 Dec 4;38:40. doi: 10.1186/s41043-019-0206-0 (PMC6892189; doi:10.1186/s41043-019-0206-0)
Supplement: Supplementary file 1 — Additional file 1. HABiT Questionnaire. [file 41043_2019_206_MOESM1_ESM.docx]

**Supplemental File – HABiT Questionnaire**

*Questions in the HABiT questionnaire have been drawn or adapted from the following sources. Details for each question and adaptations are in the chart below.*

**BIPQ: Brief Illness Perception Questionnaire**

Broadbent E, Petrie KJ, Main J, et al. The Brief Illness Perception Questionnaire. Journal of Psychosomatic Research 2006;60(6):631-37. doi: <http://dx.doi.org/10.1016/j.jpsychores.2005.10.020>

**CANRISK: Canadian Diabetes Risk Questionnaire**Robinson CA, Agarwal G, Nerenberg K. Validating the CANRISK prognostic model for assessing diabetes risk in Canada's multi-ethnic population. Chronic Dis Inj Can 2011;32(1):19-31. [published Online First: 2011/12/14]

**CCHS: Canadian Community Health Survey**

Statistics Canada. Canadian Community Health Survey - Annual Component (CCHS): Statistics Canada, 2015. <http://www23.statcan.gc.ca/imdb/p2SV.pl?Function=getSurvey&SDDS=3226>

**CDK: Chronic Disease Knowledge Questionnaire**Frantz J. A knowledge assessment questionnaire relating to risk factors for chronic disease of lifestyle for high school learners: validity and reliability. Journal of Community and Health Sciences 2015;3(1)

**CFG: Canada Food Guide**Health Canada. Eating Well with Canada's Food Guide: Health Canada, 2011.

<https://www.canada.ca/en/health-canada/services/food-nutrition/canada-food-guide/get-your-copy/eating-well-2007.html>

**CHEP: Canadian Hypertension Education Program**

Dawes MG, Kaczorowski J, Swanson G, et al. The effect of a patient education booklet and BP 'tracker' on knowledge about hypertension. A randomized controlled trial. Fam Pract 2010;27(5):472-8. doi: 10.1093/fampra/cmq048 [published Online First: 2010/07/16]

**CLADG: Canada’s Low-Risk Alcohol Drinking Guidelines**

Canadian Centre on Substance Use and Addiction. Canada's Low Risk Alcohol Drinking Guidelines. Ottawa, Ontario: Canadian Centre on Substance Use and Addiction, 2017.

<http://www.ccsa.ca/Resource%20Library/2012-Canada-Low-Risk-Alcohol-Drinking-Guidelines-Brochure-en.pdf>

**DKQ: Diabetes Knowledge Questionnaire**Garcia AA, Villagomez ET, Brown SA, Kouzekanani K, Hanis CL. The Starr County Diabetes Education Study: development of the Spanish-language diabetes knowledge questionnaire. Diabetes Care. 2001;24(1):16–21.

**EQ-5D-3L: EuroQual Five Dimensions**

Szende A, Oppe M, Devlin N. EQ-5D Value Sets: Inventory, Comparative Review and User Guide: Springer 2007

**GLTEQ: Godin Leisure-Time Exercise Questionnaire**

Godin G, Shephard, RJ. Godin Leisure-Time Exercise Questionnaire Medicine and Science in Sports and Exercise 1997: S36-S38.

**H5SQ: Health Canada 5 Stages to Quitting**

Government of Canada. 5 Stages to Quitting: Government of Canada, 2009. <http://www.hc-sc.gc.ca/hc-ps/tobac-tabac/quit-cesser/fact-fait/stages-etapes-eng.php>

**HBM: Health Belief Model Scales**

Maiman LA, Becker MH, Kirscht JP, et al. Scales for measuring health belief model dimensions: a test of predictive value, internal consistency, and relationships among beliefs. Health education monographs 1977;5(3):215-30. [published Online First: 1977/01/01]

**NVS: Newest Vital Sign-UK**~~Weiss BD, Mays MZ, Martz W, et al. Quick assessment of literacy in primary care: the newest vital sign. Ann Fam Med 2005;3(6):514-22. doi: 10.1370/afm.405 [published Online First: 2005/12/13]~~

Rowlands G, Khazaezadah N, Oteng-Ntim E, Seed P, Barr S, Weiss BD. Development and validation of a measure of health literacy in the UK: the newest vital sign. BMC Public Health 2013;13:116.

**SCDS: Stanford Chronic Disease Self-Efficacy Scales**Lorig K, Stewart A, Ritter P, González V, Laurent D, Lynch J, Outcome Measures for Health Education and other Health Care Interventions. Thousand Oaks CA: Sage Publications, 1996, pp.24-25,41-45.

**SCT: Stages of Change Theory (for smoking cessation)**
Prochaska JO, DiClemente CC. Stages and processes of self-change of smoking: toward an integrative model of change. Journal of consulting and clinical psychology 1983;51(3):390-5. [published Online First: 1983/06/01]

| **Question** | | **Source** | **Notes** |
| --- | --- | --- | --- |
| **Demographic Information** | | | |
| Identifiers:   - First Name - Middle Initial - Last Name - Date of Birth - Postal Code | | Multiple | Full date of birth , middle initial, and postal code were included to facilitate linkage with administrative databases. |
| Q1. Are you male or female?   - Male - Female | | CANRISK, CCHS |  |
| Q2. Please check off which of the following ethnic groups your biological (blood) parents belong to: (Best guess if unknown)   - Mother   - White (Caucasian)   - Aboriginal   - Black (Afro-Caribbean)   - East Asian (Chinese, Vietnamese, Filipino, Korean, etc.)   - South Asian (East Indian, Pakistani, Sri Lankan, etc.)   - Other non-white (Latin American, Arab, West Asian) - Father   - White (Caucasian)   - Aboriginal   - Black (Afro-Caribbean)   - East Asian (Chinese, Vietnamese, Filipino, Korean, etc.)   - South Asian (East Indian, Pakistani, Sri Lankan, etc.)   - Other non-white (Latin American, Arab, West Asian) | | CANRISK |  |
| Q3. What is the highest level of education that you have completed?   - Some high school or less - High school diploma - Some college or university - University or college degree | | CANRISK |  |
| Q4. What is your current marital status? *Check the one best answer.*   - Married - Living Common-law - Widowed - Separated - Divorced - Single, never married | | CHEP, CCHS | Question text: CHEP  Response options: CHEP, but some response options were split to match CCHS. |
| **Health Status and Quality of Life** | | | |
| Q5. In general, would you say your health is:   - Excellent - Very good - Good - Fair - Poor | | CCHS |  |
| By placing a tick in one box in each group below, please indicate which statements best describe your own health state today.  Q6. Mobility   - I have no problems in walking about - I have some problems in walking about - I am confined to bed   Q7. Self-Care   - I have no problems with self-care - I have some problems with washing and dressing myself - I am unable to wash or dress myself   Q8. Usual Activities *(e.g. work, study, housework, family or leisure activities)*   - I have no problems with performing my usual activities - I have some problems with performing my usual activities - I am unable to perform my usual activities   Q9. Pain/Discomfort   - I have no pain or discomfort - I have moderate pain or discomfort - I have extreme pain or discomfort   Q10. Anxiety/Depression   - I am not anxious or depressed - I am moderately anxious or depressed - I am extremely anxious or depressed | | EQ-5D-3L |  |
| To help people say how good or bad a health state is, we have drawn a scale (rather like a thermometer) on which the best state you can imagine is marked 100 and the worst state you can imagine is marked 0.  Q 11. We would like you to indicate on this scale how good or bad your own health is today, in your opinion. Please do this by drawing a line from the box below to whichever point on the scale indicates how good or bad your health state is today.  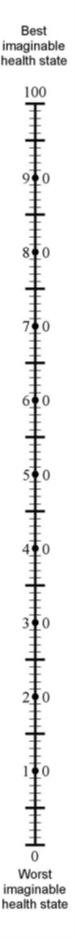  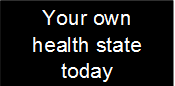 | | EQ-5D-3L | Note: This visual analog scale should be 20cm in length when printed. |
| **Knowledge and Risk Factors** | | | |
| Q12. Please try to answer the questions below by circling your answer to the statements below.  [Response options for all items below are: Definitely True, Maybe True, Not Sure or Don’t Know, Maybe False, and Definitely False] | | Multiple Sources (see each item) | The response options were “True, False, and Don’t Know” in CHEP and have been expanded to a 5-point scale. |
|  | - High blood pressure is a risk factor for heart attacks and strokes | CHEP |  |
|  | - Diabetes is a risk factor for heart attacks and strokes |  | Replicated previous item for diabetes. |
|  | - High blood pressure can cause other serious health problems | CHEP |  |
|  | - Diabetes can cause other serious health problems |  | Replicated previous item for diabetes. |
|  | - High blood pressure becomes more common as people get older | CHEP | Adapted from, “High BP becomes more common with aging.” |
|  | - Diabetes becomes more common as people get older |  | Replicated previous item for diabetes. |
|  | - You can tell if you have high blood pressure (without a blood pressure cuff) because you will probably feel unwell | CHEP |  |
|  | - Lifestyle changes such as stopping smoking and weight loss can decrease blood pressure | CDK |  |
|  | - The following blood pressure is considered to be high: 140/90 | CDK | Adapted from 130/80. |
|  | - In the general population the following things can contribute to people having high blood pressure:   - having a stressful lifestyle   - drinking too much alcohol   - eating too much salt | CHEP | These are considered three questions. |
|  | - You are at risk of developing diabetes if you are obese. | CDK | Adapted for diabetes from the original question, “You are at risk of getting a stroke if you are obese.” |
|  | - Eating too much sugar and other sweet foods is a cause of diabetes. | CHEP, DKQ, CDK |  |
|  | - People who have family members with diabetes have an increased risk of developing diabetes. | CHEP |  |
|  | - Diabetes can be cured. | CHEP, DKQ, CDK |  |
|  | - High blood pressure can be treated by exercise and weight loss. | CDK | Adapted from, “Hypertension can be treated with medication, exercise, & weight loss.” |
|  | - To reduce the risk of diabetes you need to eat well and exercise regularly. | CDK | Adapted from, “To reduce the risk of stroke you need to eat well and exercise regularly.” |
|  | - The recommended blood pressure for most adults is less than 120/80. | CHEP | Adapted from, “What is the recommended target (ideal) blood pressure for most adults?” and provided 5 possible response options (less than 150/90mmHg; Less than 140/90mmHg; Less than 130/85mmHg; etc...” |
| Q13. Have you ever had your blood pressure taken?   - Yes - No | | CCHS |  |
| Q14. When was the last time?   - Less than 6 months - 6 months to less than 1 year ago - 1 year to less 2 years ago - 2 years to less than 5 years ago - 5 or more years ago | | CCHS |  |
| Q15. Have you ever been told by a doctor or nurse that you have high blood pressure OR have you ever taken high blood pressure pills?   - Yes - No - Don’t know | | CANRISK |  |
| Q16. Have you have your cholesterol levels checked in the past 2 years?   - Yes - No - Don’t know | | CHEP | Adapted from, “…checked regularly?” |
| Q17. Have you have your blood sugar levels checked in the past 2 years?   - Yes - No - Don’t know | | CHEP | Adapted from, “….checked regularly?” |
| Q18. Do you know what your most recent blood pressure reading was?   - Yes   - What was it? (___/___mmHg)   - Date of measurement? (date) - No - Don’t know – wasn’t told | | CHEP |  |
| Q19. Have you ever been found to have high blood sugar either from a blood test, during an illness, or during pregnancy?   - Yes - No - Don’t know | | CANRISK |  |
| Q20. Have you ever given birth to a large baby weight 9 pounds (4.1 kg) or more? (*Skip if male*)   - Yes - No - Don’t know | | CANRISK |  |
| Q21. Have any of your blood relatives ever been diagnosed with diabetes? *Check all that apply.*   - Mother - Father - Brothers/Sisters - Children - No/Don’t know - Other | | CANRISK |  |
| Q22. Do you have any of the following health problems? *Check all that apply.*   - Heart Problems - High Blood Pressure - High Cholesterol - Stroke - Diabetes - None of the above | | CHEP | Removed some response options that were not relevant to the CP@clinic program. |
| Q23. How tall are you and how much do you weight?   - Weight (pounds or kilograms) - Height (feet and inches; centimetres; don’t know) | | CANRISK |  |
| Q24. Using a tape measure, place it around your waist at the level of your belly button. *Measure after breathing out (do not hold your breath) and write your results on the line below. Then check the box that contains your measurement.*   - Measured: Waist circumference (inches or centimeters) - Estimated: Pant size | | CANRISK | Pant size is not an option in CANRISK, but was added to help impute missing data. |
| Q25. Do you usually do some physical activity such as brisk walking for at least 30 minutes each day? *This activity can be done while at work or home.*   - Yes - No | | CANRISK |  |
| Q26. During a typical 7-Day period (a week), how many times on the average do you do the following kinds of exercise for more than 15 minutes during your free time (write on each line the appropriate number).   - Mild exercise (minimal effort) – e.g. yoga, stretching, low intensity strength exercises, walking a dog, household cleaning, laundry, gardening - Moderate exercise (not exhausting) - e.g., brisk walking, slow stair climbing, easy bicycling, water - aerobics, low impact group exercise classes, cultural dancing - Strenuous exercise (heart beats rapidly) - e.g., vigorous swimming, vigorous bicycling, fast stair climbing, strenuous group exercise, running, fast dancing | | GLTEQ | Examples changed to activities more relevant to seniors based on the published compendium of METs for PA activities. |
| Q27. In a typical day of the week, how much time did you usually spend on a computer, including playing computer games and using the Internet?   - More than 12 hours - 10-12 hours - 7-9 hours - 3-6 hours - Less than 3 hours - Not applicable | | CCHS |  |
| Q28. In a typical day of the week, how much time did you usually spend watching television or videos?   - More than 12 hours - 10-12 hours - 7-9 hours - 3-6 hours - Less than 3 hours - Not applicable | | CCHS |  |
| Q29. In a typical day of the week, how much time did you usually spend sitting down reading, not counting at work or at school?   - More than 12 hours - 10-12 hours - 7-9 hours - 3-6 hours - Less than 3 hours - Not applicable | | CCHS |  |
| Now, some questions about the foods you eat.  Q30. How often do you eat vegetables or fruit?   - Every day - Not every day; if so, how often do you eat them in a week?   - 4-5 times a week   - 2-3 times a week   - Once a week   - Less than once a week | | CANRISK, CHEP | CANRISK only provides two response options: every day and not every day.  The response options were further broken down to match CHEP. |
| Q31. How many pieces of fruit/portions of vegetables do you normally eat per day?   - 7 or more - 5 – 7 per day - 3 – 4 per day - 1 – 2 per day - None | | CHEP, CFG | Original response options: 5 or more; 3-4 per day; 1-2 per day; None.  The response options were expanded to match the Canada Food Guide. |
| Q32. Do you monitor your food intake to reach or maintain a healthy weight?   - All the time - Frequently - Sometimes - Rarely - Not at all | | CHEP | Response options changed from “Yes/No” to a 5-point scale.. |
| Q33. How often do you eat bread, cereal, potatoes, rice or pasta?   - As a part of every meal - As part of 1 or 2 meals each day - 1 to 3 times per week - 4 to 6 times per week - Never or hardly | | CHEP |  |
| Q34. How often do you eat fatty food, *e.g. fried food or savoury snacks (ie. chips or peanuts)*   - Never - 2-3 times a month - 1-2 times a week - 3-4 times a week - Every day | | CHEP | Q34 and Q35 were originally a single question (fatty food and sugary food) and were split into two questions in the HABiT. |
| Q35. How often do you eat sugary food, *e.g. cookies, chocolate?*   - Never - 2-3 times a month - 1-2 times a week - 3-4 times a week - Every day | | CHEP | See question 34. |
| Q36. How often do you add salt to your food at the table or in cooking?   - Never - Rarely - Sometimes - Often - Always | | CHEP | Original response options were: Never/Rarely, Sometimes, and Always. Expanded to a 5-point scale. |
| Q37. Are you a/an: *(Check the one best answer)*   - Daily smoker - Occasional smoker - Former smoker who quit – **Go to Q39** - Never smoked – **Go to Q41** | | CHEP, CCHS | Merged questions from both sources |
| Q38. If you are a smoker, do you:   - Have no plans to quit - Have thought about quitting - Have a plan to quit smoking - Initiated the plan to quit smoking - Never smoked | | SCT, H5SQ | Question developed from Stage of Change theory for smoking cessation and Health Canada’s Five Stages to Quitting |
| Q39. If you are a current or ex-smoker, how much do you now, or did you previously, smoke on average per day?  ___ cigarettes per day  ___ cigars per day  ___ pipes per day | | CHEP |  |
| Q40. If you are an ex-smoker, how many years ago did you stop? *(If less than one year, please answer with a zero)*  ___ years | | CHEP |  |
| Now, some questions about your alcohol consumption.  When we use the word ‘drink’ it means:   - Beer (5% alcohol content), 341 ml or 12 oz. - Wine (12% alcohol content) 142 ml or 5oz. - Wine cooler/Cider (5% alcohol content) 341ml or 12oz. - Alcohol (rye, rum, gin) (40% alcohol content) 43ml or 1.5oz | | CCHS |  |
| Q41. How many drinks of alcohol do you drink in an average week? *Check the one best answer.*   - None - 1-5 - 6-10 - 11-15 - More than 15 | | CHEP, CLADG | Adapted the response options from CHEP to match Canada’s Low-Risk Alcohol Drinking Guidelines. |
| Q42. How often in the past 12 months have you had 5 or more drinks on one occasion?   - Never or less than once a month - Once a month - 2 to 3 times a month - Once a week - More than once a week | | CCHS |  |
| Now a few questions about the stress in your life.  Q43. In general, how would you rate your ability to handle unexpected and difficult problems, for example, a family or personal crisis? Would you say your ability is:   - Excellent - Very good - Good - Fair - Poor | | CCHS |  |
| Q44. In general, how would you rate your ability to handle the day-to-day demands in your life, for example, handling work, family and volunteer responsibilities? Would you say your ability is:   - Excellent - Very good - Good - Fair - Poor | | CCHS |  |
| Q45. Thinking about stress in your day-to-day life, which of the following contribute to feelings of stress you may have? *Check all that apply.*   - Time pressures/not enough time - Own physical health problem or condition - Own emotional or mental health problem or condition - Financial situation (e.g., not enough money, debt) - Own work situation (e.g., hours of work, working conditions) - School - Employment status (e.g., unemployment) - Caring for - own children - Caring for - others - Other personal or family responsibilities - Personal relationships - Discrimination - Personal and family’s safety - Health of family members - Other - Specify ________________________ - Nothing | | CCHS |  |
| **Health Utilization and Access** | | | |
| Q46. Overall, how would you rate the availability of health care services in your community?   - Excellent - Very good - Good - Fair - Poor | | CCHS | Adapted to add “Very good” as a response option. |
| Q47. Where do you go to get information about keeping your heart healthy? *Please put a checkmark in the box for up to 3 useful sources:*   - Family member or friend - Media (print ads, television) - Educational brochures or pamphlets - Internet - My doctor or nurse - My pharmacist - Walk-in Clinic - Blood pressure booklet & ‘Tracker’ - Blood pressure screening provided by paramedics  in our building - Other, Specify: __________________ | | CHEP | Adapted to add “Blood pressure screening provided by paramedics in our building” to include the intervention being evaluated.  Also split the question since it was originally double-barreled with “heart healthy and preventing diabetes.” |
| Q48. Where do you go to get information about preventing diabetes? *Please put a checkmark in the box for up to 3 useful sources:*   - Family member or friend - Media (print ads, television) - Educational brochures or pamphlets - Internet - My doctor or nurse - My pharmacist - Walk-in Clinic - Blood pressure booklet & ‘Tracker’ - Blood pressure screening provided by paramedics  in our building - Other, Specify: __________________ | | CHEP | See notes about Q47. |
| Q49. Is there a place that you usually go to when you are sick or need advice about your health?   - Yes - No | | CCHS |  |
| Q50. If yes, what kind of place is it? *Check all that apply.*   - Doctor’s office - Community health centre / CLSC - Walk-in clinic - Telephone health line (for example, Telehealth Ontario, Info-Santé) - TeleCare - Hospital emergency room - Hospital outpatient clinic - Other - Specify ______________ | | CCHS |  |
| Q51. Do you have a regular family doctor?   - Yes - No – **Go to Question 53** | | CCHS |  |
| Q52. How many times have you visited your regular family doctor in the last 12 months?  _____ times | | CHEP | Adapted from original question, “How many times have you been admitted to the hospital (and stayed overnight) in the last 12 months?” |
| Q53. How many times have you visited you called EMS (ambulance) in the last 12 months?  _____ times | | CHEP | See Q52. |
| Q54. How many times have you visited the walk-in clinic in the last 12 months?  _____ times | | CHEP | See Q52. |
| Q55. Has there been a recent time that you did not feel well and did not get care? Why didn’t you get care?   - Not applicable (sought out care) - Not available - in the area - Not available - at time required (e.g. doctor on holidays, inconvenient hours) - Waiting time too long - Felt would be inadequate - Cost - Too busy - Didn’t get around to it / didn’t bother - Decided not to seek care - Other – Specify _____________ | | CCHS |  |
| **Perceived Concern and Understanding of Risk** | | | |
| Q56. How concerned are you about high blood pressure?  [Response is noted on a 7-point scale from “Not at all concerned” to “Extremely concerned”] | | BIPQ, HBM | Changed the BIPQ question from “…about your illness?” to “… about high blood pressure?” |
| Q57. How concerned are you about diabetes?  [Response is noted on a 7-point scale from “Not at all concerned” to “Extremely concerned”] | | BIPQ, HBM | Changed the BIPQ question from “…about your illness?” to “…about diabetes?” |
| Q58. How well do you feel you understand your risk of high blood pressure?  [Response is noted on a 7-point scale from “Do not understand” to “Completely understand”] | | BIPQ, HBM | Changed the BIPQ question from “…understand your illness?” to “… understand your risk of high blood pressure?” |
| Q59. How well do you feel you understand your risk of diabetes?  [Response is noted on a 7-point scale from “Do not understand” to “Completely understand”] | | BIPQ, HBM | Changed the BIPQ question from “…understand your illness?” to “…understand your risk of diabetes?” |
| Q60. How important is it for you to increase your intake of fruit and vegetables in your diet?  [Response is noted on a 7-point scale from “Not at all important” to “Extremely important”] | | HBM |  |
| Q61. How important is it for you to decrease your intake of foods high in salt?  [Response is noted on a 7-point scale from “Not at all important” to “Extremely important”] | | HBM |  |
| Q62. How important is it for you to increase your physical activity?  [Response is noted on a 7-point scale from “Not at all important” to “Extremely important”] | | HBM |  |
| Q63. Is there anything you intend to do to improve your physical health in the next year?   - Yes - No | | CCHS |  |
| Q64. If yes, what is that?   - Start / Increase exercise, sports / physical activity - Lose weight - Change diet / improve eating habits - Increase fruit and vegetable intake - Quit smoking / reduce amount smoked - Drink less alcohol - Reduce stress level - Receive medical treatment - Other, Please specify __________________ | | CCHS | Adapted by removing the response option, “Take vitamins.” |
| Q65. If you currently smoke daily or occasionally, are you seriously considering quitting smoking within the next 6 months?   - Yes - No - Not applicable | | CCHS |  |
| How confident are you, that in the next year, you will be able to:  [Response options for Q66 – Q70 are 7-point scales from “Not at all confident” to “Extremely confident”]  Q66. Improve your physical activity  Q67. Increase your fruit and vegetable intake  Q68. Reduce your alcohol intake  Q69. Quit smoking  Q70. Reduce stress | | SCDS | These questions followed the format of the SCDS scales. The response options were changed from a 6-point scale to a 7-point scale to match the rest of scales in the HABiT, but the anchors remained the same. |
| **Health Literacy** | | | |
| *For all questions below, show the laminated nutrition label card to the respondent:*  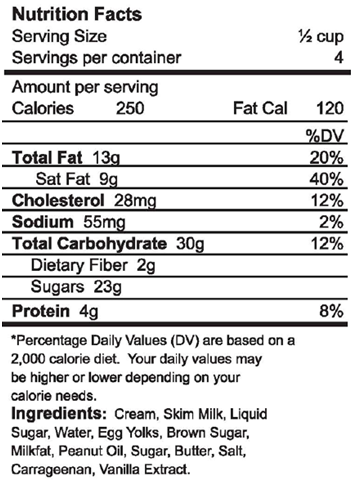 | | NVS |  |
| Q71. How many calories (kcal) will you eat if you eat the whole container?   - 1,000 KCAL - 1,000 CALORIES - Any other answer - Don’t know - Refused | | NVS |  |
| Q72. If you are advised to eat no more than 60 grams of carbohydrate for dessert, what is the maximum amount of ice cream you could have?   - Two servings (or anything up to 2 servings) - Half the container (or any amount up to half the container) - 200 ml (or any amount up to 200 ml) - Any other answer - Don’t know - Refused | | NVS |  |
| Q73. Imagine that your doctor advises you to reduce the amount of saturated fat in your diet. You usually have 42g of saturated fat each day, some of which comes from one serving of ice cream. If you stop eating ice cream, how many grams of saturated fat would you be eating each day?   - 33 g - Any other answer - Don’t know - Refused | | NVS |  |
| Q74. If you usually eat 2500 calories a day, what percentage of your daily calorie (kcal) intake will you get if you eat one serving of ice cream?   - 1/10 (one tenth) - 10% - Any other answer - Don’t know - Refused | | NVS |  |
| **READ OUT:** Imagine that you are allergic to the following substances: penicillin, peanuts, latex, gloves, and bee stings. | | NVS |  |
| Q75. Is it safe for you to eat this ice cream?   - Yes – **Go to Q77** - No - Don’t know – **Go to Q77** - Refused – **Go to Q77** | | NVS |  |
| Q76. Why not?   - Because it contains peanut oil/peanuts/nuts – **Go to Q77** - Because you might have an allergic reaction - Any other answer – **Go to Q77** - Don’t know – **Go to Q77** - Refused – **Go to Q77** | | NVS |  |
| Q77. Why would you have an allergic reaction?   - Because it contains peanut oil/peanuts/nuts - Any other answer - Don’t know - Refused | | NVS |  |
